# Trustworthy Breast Ultrasound Image Semantic Segmentation Based on Fuzzy Uncertainty Reduction

**DOI:** 10.3390/healthcare10122480

**Published:** 2022-12-08

**Authors:** Kuan Huang, Yingtao Zhang, Heng-Da Cheng, Ping Xing

**Affiliations:** 1 Department of Computer Science and Technology, Kean University, Union, NJ 07083, USA; 2 School of Computer Science and Technology, Harbin Institute of Technology, Harbin 150001, China; 3 Department of Computer Science, Utah State University, Logan, UT 84322, USA; 4 Ultrasound Department, The First Affiliated Hospital of Harbin Medical University, Harbin 150001, China

**Keywords:** fuzzy logic, uncertainty reduction, semantic segmentation, breast ultrasound (BUS) image

## Abstract

Medical image semantic segmentation is essential in computer-aided diagnosis systems. It can separate tissues and lesions in the image and provide valuable information to radiologists and doctors. The breast ultrasound (BUS) images have advantages: no radiation, low cost, portable, etc. However, there are two unfavorable characteristics: (1) the dataset size is often small due to the difficulty in obtaining the ground truths, and (2) BUS images are usually in poor quality. Trustworthy BUS image segmentation is urgent in breast cancer computer-aided diagnosis systems, especially for fully understanding the BUS images and segmenting the breast anatomy, which supports breast cancer risk assessment. The main challenge for this task is uncertainty in both pixels and channels of the BUS images. In this paper, we propose a Spatial and Channel-wise Fuzzy Uncertainty Reduction Network (SCFURNet) for BUS image semantic segmentation. The proposed architecture can reduce the uncertainty in the original segmentation frameworks. We apply the proposed method to four datasets: (1) a five-category BUS image dataset with 325 images, and (2) three BUS image datasets with only tumor category (1830 images in total). The proposed approach compares state-of-the-art methods such as U-Net with VGG-16, ResNet-50/ResNet-101, Deeplab, FCN-8s, PSPNet, U-Net with information extension, attention U-Net, and U-Net with the self-attention mechanism. It achieves 2.03%, 1.84%, and 2.88% improvements in the Jaccard index on three public BUS datasets, and 6.72% improvement in the tumor category and 4.32% improvement in the overall performance on the five-category dataset compared with that of the original U-shape network with ResNet-101 since it can handle the uncertainty effectively and efficiently.

## 1. Introduction

Medical imaging is the most important approach in the early detection and diagnosis of diseases. A trustworthy computer-aided diagnosis (CAD) system is designed to assist doctors and radiologists in making a diagnostic decision. Image segmentation is one of the most important steps in a CAD system. It can detect lesions and separate them from the background. The accuracy of segmentation can affect if the CAD system is trustable or not. Image segmentation has been applied to computed tomography (CT) imaging for lung and nasopharyngeal cancer [1,2], magnetic resonance (MR) imaging for breast, musculoskeletal and brain [3,4], chest and dental X-ray imaging [5,6], and ultrasound imaging [7]. Before the advance of the deep convolutional neural network, medical image segmentation methods were based on classic machine learning and computer vision methods such as watershed-based method [1], thresholding method [8], clustering method [9], active contour model [10], Markov model [11], etc.

**Figure 1:**
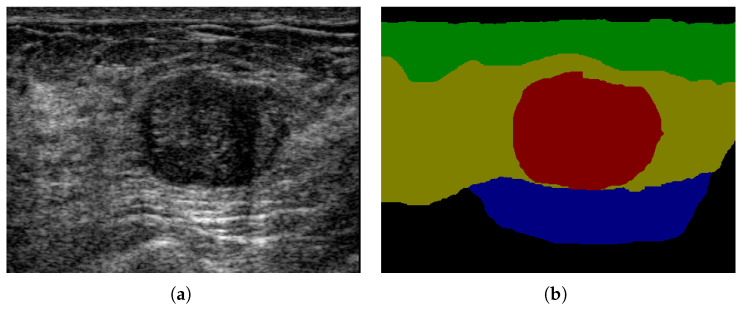
Breast anatomy: (**a**) BUS image; (**b**) ground truth; green: fat layer, yellow: mammary layer, blue: muscle layer, red: tumor, and black: background.

Comparing with CT, MR, X-Ray imaging, ultrasound imaging is harmless, low cost, and potable. Breast ultrasound (BUS) imaging is one of the most important modalities for breast cancer early detection [12,13]. However, BUS images are usually in low contrast, and poor quality and have inherent speckle noise and shadows [14]. It is critical to develop computer-aided diagnosis systems for breast ultrasound images, especially for breast anatomy segmentation (multi-category BUS semantic segmentation). The location relation between breast tissues and the tumor can provide important context information in breast cancer diagnosis (shown in Figure 1). For example, the tumor region (red) is much more likely located in the mammary layer (yellow) than in other layers. The breast anatomy can also provide important information for breast density calculation which has high correlation with cancer risk [15]. There are only few researches in multi-category BUS semantic segmentation because most BUS image datasets only contain ground truths for tumors. In [16], U-Net was applied to BUS image segmentation with three categories: tumor, mammary layer, and background. The location relation between the mammary layer and the tumor was employed to refine the segmentation results. An encoder-decoder network with deep boundary regularized constraint, and adaptive domain transfer was proposed [17] to segment four layers in BUS images. In [18], a deep learning method based on a self-attention mechanism was proposed for breast anatomy layer segmentation.

Although there are some segmentation networks for BUS image [19,20,21,22] which increase the performance of BUS image segmentation, there are three main challenges in breast ultrasound (BUS) image segmentation: (1) The edges of the lesion area in BUS images are generally blurred (as shown in Figure 1a). (2) The background regions in BUS images contain similar intensity to the lesion area. (3) The boundaries of different breast tissue layers are hard to classify, which is a disadvantage for the segmentation of breast tissues. Those challenges are the uncertainty in BUS images. Meanwhile, deep learning algorithms also contain uncertainty. In [23,24], it shows epistemic and aleatoric uncertainty in deep learning architecture and medical images. The entropy [24] and hierarchical resolution segmentation [23] are used to estimate the uncertainty [23]. Attention mechanisms in convolutional neural networks demonstrate that different pixels and channels in a feature map contain different importance degrees in making the final classification decision. They can provide context information to generate novel features and present noise in the original feature map. Attention mechanisms can also reduce the random uncertainty in deep learning methods in pixels and channels of the convolutional features by spatial-wise and channel-wise attention mechanisms [25,26]. The uncertainty in the pixels and channels measures the difficulty in classifying the pixels and channels into different categories. However, attention mechanisms cannot handle the non-random/statistical uncertainty. Fuzzy logic methods [27,28] are used to handle non-random uncertainty in many classic machine learning and deep learning algorithms.

In order to increase the accuracy of BUS image segmentation in CAD systems, and take the advantages of both attention mechanisms and fuzzy logic, two novel fuzzy attention mechanisms: the spatial-wise and channel-wise fuzzy blocks are added to the classic U-shape network with a ResNet-101 network structure, and the Spatial and Channel-wise Fuzzy Uncertainty Reduction Network (SCFURNet) is proposed to reduce uncertainty and noise in BUS images and to conduct the semantic segmentation. The major contributions of this research are:

1. The proposed spatial-wise fuzzy blocks (SFBs) are applied to measure and reduce the spatial uncertainties (spatial dimension) in convolutional feature maps, and the proposed channel-wise fuzzy blocks (CFBs) are proposed to handle the channel uncertainties (channel dimension) in convolutional feature maps.
2. A novel membership function in deep learning is designed. Membership functions in fuzzy blocks are defined by 1×1 convolutional operator with a Sigmoid activation function to increase the non-linearity of the membership function.
3. A novel fuzzy logic uncertainty measurement method is proposed. Fuzzy entropy [29,30,31] calculated by the memberships of different categories are utilized to measure the uncertainties for pixels and channels. Uncertain pixels and channels are those with higher fuzzy entropies (details will be discussed in Section 3).

The paper is organized as follows: We briefly review the related works in Section 2. Section 3 introduces the proposed spatial and channel-wise fuzzy uncertainty reduction method. Section 4 shows the semantic segmentation results on four datasets and compares the proposed method with state-of-the-art methods. Discussions based on experimental results are presented in Section 5. The conclusions are in Section 6.

## 2. Related Works

### 2.1. BUS Image Segmentation

Classic machine learning and computer vision approaches have been applied to BUS image segmentation and classification [32,33]. A gray-level thresholding method was proposed to find the region of interests (ROIs) of tumors, and the area growing method was employed for tumor segmentation on ROIs [8]. A method based on k-means clustering [34] was reported. The classic k-means clustering was enhanced by Ant Colony Optimization (ACO) in initializing cluster centroid, and a regularization term was added to the k-means clustering function to increase the stability of the clustering method. The non-deep learning methods apply classic machine learning algorithms and computer vision methods to BUS image segmentation. The performances are depended on datasets and the manually extracted features, such as texture, gray-level intensity, frequency features, etc.

Recently, deep convolutional neural network-based approaches have been widely utilized in image semantic segmentation. Semantic segmentation approaches are frequently based on deep convolutional neural networks because they can learn features automatically. Such characteristic avoids selecting features manually and reduces noise effect in some cases. There are also researches in BUS image semantic segmentation using deep learning. Deep learning based semantic segmentation of BUS image can provide a better understanding of BUS image and the category information of each pixel, which is important in trustworthy CAD systems. However, most of the BUS image semantic segmentation methods are only focus on tumor area and background area. In [35], fully convolutional network (FCN) [36] was utilized for tumor segmentation in BUS images. Three networks were utilized and compared with LeNet [37], U-Net [38], and a pre-trained FCN with AlexNet [39]. A stacked denoising auto-encoder (SDAE) was employed to diagnose breast ultrasound lesions and lung CT nodules in [40]. In [41], a deep learning approach was specifically designed for small tumors. Different sizes of convolutional kernels were employed for convolutional blocks to detect tumors. Multi-category BUS semantic segmentation is important for breast cancer diagnosis. The experiment results show that deep learning methods achieve good results on BUS image semantic segmentation. However, deep learning methods require a great number of training samples. Moreover, most of the previous deep learning methods do not consider the non-random uncertainty inside deep learning architectures.

### 2.2. Attention Mechanisms

Attention mechanism in convolutional neural networks is popularly used [42] to reduce noise and uncertainty. It assigns the weights to pixels or channels of feature maps to express the importance. In [43], a spatial-wise attention gate was proposed in the decoder of U-Net. The encoder and decoder information were combined to calculate a weight tensor before concatenating the encoder-feature map and the decoder information. The weight tensor multiplied with the encoder-feature map. The attention coefficients were bigger in the target areas than those in the background, and the results were better than that of the original U-Net. In [44], Hu et al. proposed a channel-wise attention mechanism, Squeeze-and-Excitation Networks (SE-Nets). A convolutional operator transformed the feature map in each convolutional block. Then, in each channel, a global average pooling was performed to calculate the mean value of each channel. The results were used as the weight values for the channels in the original feature map. The SE block in SE-Nets was applied to network architectures such as VGG-16, ResNet-101, etc., and achieved good improvement. In [45], both spatial-wise and channel-wise attention mechanisms were applied to the image caption. The network structure followed VGG-19 [46] and ResNet-152 [47]. In each convolutional block, the weights of spatial-wise attention were based on the original feature map and last sentence context information. The mean value for each channel of the original feature map and last sentence context information was used to calculate the channel-wise attention weights. Another spatial and channel-wise attention FCN [48] was proposed for crowd counting. The network structure followed VGG-16 [46] architecture. The spatial-wise and channel-wise attention weights were computed by the original feature map in the same convolutional block. The original feature map was inputted to three 1×1 convolutional kernels in the spatial-wise attention. Then, reshaping and transposing operators were applied to the outputs of the 1×1 convolutional kernels to obtain three new features. For channel-wise attention weights, only one 1×1 convolutional kernel was utilized. Then, it was reshaped and transposed to three different sizes. The attention weights were computed by multiplying and adding three different size features.

### 2.3. Fuzzy Logic in Deep Learning

The attention mechanism can reduce uncertainty and noise in convolutional feature maps; however, uncertainties are not caused by randomness only and cannot be handled by statistics, probabilities, and attention mechanisms well. Fuzzy logic has been utilized to handle the uncertainties successfully in image processing. A fuzzy clustering method, fuzzy c-means clustering [49] was applied to image segmentation. The fuzzy clustering method achieved better performance than the non-fuzzy version. A fuzzy contrast enhancement method [50] was proposed. The maximum entropy principle was utilized to map the image from the feature domain to the fuzzy domain. A fuzzy cellular automata framework [51] was proposed to handle the uncertainty in BUS images. The cellular automata results were transformed into the fuzzy domain, and a majority voting strategy was utilized. In order to remove speckle noise and inhomogeneous echoes, two kinds of texture features were involved. In [52], an adaptive fuzzy neural network was proposed. Input samples were mapped into the fuzzy domain by a trainable Gaussian membership function. Huang et al. [53] proposed a fuzzy logic-based FCN for multi-layer BUS image segmentation and a conditional random field-based method for post-processing. However, the fuzzy logic operator was only applied to the input image and the first convolutional feature. Also, this method only solved uncertainty in the pixel dimension.

## 3. Methods

### 3.1. Overview

**Figure 2:**
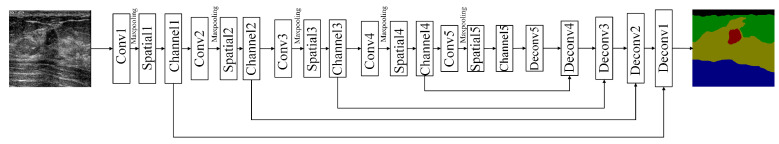
The proposed network structure.

Figure 2 illustrates the entire network structure for the proposed SCFURNet. The proposed SCFURNet is based on a U-Shape network that contains an encoder branch for feature extraction and a decoder branch for segmentation. The encoder network contains five convolutional blocks, and the decoder network contains five deconvolutional blocks. SCFURNet consists of the U-Shape network and two novel components: (1) the spatial-wise fuzzy block (SFB) and (2) the channel-wise fuzzy block (CFB). We add five SFBs and five CFBs to the five convolutional blocks in the encoder network. The output for each convolutional block is processed by an SFB and a CFB sequentially and then inputted to the next convolutional block. This process indicates that the SFBs and the CFBs reduce the uncertainty of convolutional features from five convolutional blocks. Convolutional blocks in VGG-16 [46] and ResNet-101 [47] network structures are utilized as the encoder in the proposed network for comparison. Two different kinds of convolutional blocks in VGG-16 and ResNet-101 are used as the encoder network to compare the performance of different kinds of convolutional blocks and show the effectiveness of the proposed SFB and CFB in different kinds of convolutional blocks. The SFB and the CFB will be explained in detail in Section 3.2 and Section 3.3, respectively.

### 3.2. Spatial-Wise Fuzzy Block

**Figure 3:**
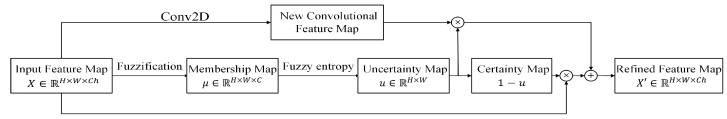
Spatial-wise fuzzy block.

An SFB is utilized to calculate the uncertainty of each pixel and reduce the uncertainty in each convolutional feature map. In the SFB, there are three major components: fuzzification, uncertainty representation, and uncertainty reduction. The flowchart of the SFB is shown in Figure 3.

#### 3.2.1. Fuzzification

Each input node from the original feature map is mapped to the fuzzy domain by membership function f(·):

(1) $$\mu_{i}=f\left(x_{i}\right)$$

where f(·) represents the membership function; $x_{i}$ represents the input node *i* (here it is a pixel in the input feature map $X\in R^{H\times W\times Ch}$, *H*, *W*, and Ch represent the height, width, and the number of channels of the feature map, respectively); $\mu_{i}\in R^{C}$ represents the memberships of the input node, where *C* is the number of categories. In some researches [50,52], f(·) was an S-shape function, Sigmoid function, or Gaussian function.

In this research, the original features are transformed into fuzzy domain by the trainable Sigmoid membership function:

(2) $$\mu_{ir}=\frac{1}{1+\exp(\alpha_{ir}x_{i}+\beta_{ir})}$$

where $x_{i}\in R^{Ch}$ is the $i^{\mathrm{th}}$ pixel in the input feature map. $\alpha_{ir}\in R^{Ch}$ and $\beta_{ir}\in R$ are two trainable parameters for the trainable Sigmoid function, and $\mu_{ir}\in R$ represents the membership in the $r^{\mathrm{th}}$ category.

The Sigmoid membership function can be performed by a 1×1 convolutional operation. In this research, two 1×1 convolutional layers are used as the membership function:

(3) μ=Conv1×1(Conv1×1(X))

where $\mu \in R^{H\times W\times C}$ represents the spatial membership map for input feature map *X*; Conv1 × 1 represents the 2-dimensional (2D) 1×1 convolutional layer; both convolutional layers contain *C* kernels. Here, two-layer 1×1 convolution is utilized, and it can enable the membership to fit different categories. $\mu_{i}\in \mu =\left[\mu_{i1},\mu_{i2},...,\mu_{iC}\right]$ is defined as the membership vector of pixel *i* in *X*. The outputs are normalized by the Soft-max function.

#### 3.2.2. Uncertainty Representation

Fuzzy logic is used to handle uncertainty. The memberships express the degrees that the pixel belongs to the categories and can measure the uncertainty. There is an observation for uncertain pixels: it is hard to assign to a category if a pixel contains similar memberships of different categories. Fuzzy entropy is utilized to reflect such observation, i.e., an uncertain pixel is defined as a pixel with high fuzzy entropy (close to 1), and a certain pixel is defined as a pixel with low fuzzy entropy (close to 0).

For membership vector $\mu_{i}$, the fuzzy entropy is defined as below [54]:

(4) $$H\left(\mu_{i}\right)=-\frac{1}{\log C}\times \sum_{r=1}^{C}\mu_{ir}\log\mu_{ir}$$

where *C* represents category number; and $\mu_{ir}$ represents the membership of category *r*. If the memberships for all categories are the same ($\mu_{ir}=\frac{1}{C}$), the entropy is the highest ($H(\mu_{i})=1$). It is hard to assign a category when the memberships for all categories are the same.

In the SFB, the memberships are utilized to calculate the fuzzy entropy as Equation (5):

(5) $$u_{i}=H\left(\mu_{i}\right)$$

where $u_{i}$ is the uncertainty degree of pixel *i*, which is in [0, 1]. 0 represents low uncertainty, and 1 represents high uncertainty. Every pixel in the input feature map contains the corresponding uncertainty degree. The uncertainty degrees for all pixels consist of the uncertainty map. The uncertainty map has the same size as the input feature map.

#### 3.2.3. Uncertainty Reduction

If the uncertainty degree $u_{i}$ is close to 1, the feature for pixel *i* generated in the convolutional block is uncertain. If the uncertain degree $u_{i}$ is close to 0, the feature for pixel *i* obtained in the convolutional block is useful for the final decision. The features of uncertain pixels should reduce weight in the novel feature map. The features will replace the uncertain pixels to reduce the uncertainty.

Shown as Figure 3, the uncertainty map ($u\in R^{H\times W}$) which consists of uncertainty degrees ($u_{i}$) in Equation (5) is utilized as the weight in the combination of the input feature map and a novel feature map; 1−u represents the certainty map:

(6) $$X^{\prime }=\left(\mathrm{Conv}2D\left(X\right)⊗u\right)⊕X⊗\left(1-u\right)$$

where $X^{\prime }\in R^{H\times W\times Ch}$ represents the refined feature map after reducing uncertainty; Conv2D represents a 2D 3×3 convolutional layer with Ch kernels, stride = 1 and padding = 1; ⊗ represents the pixel-wise multiplication between *u* or 1−u and each channel of Conv2D(*X*) or *X*, and ⊕ represents the pixel-wise summation of matrices. This uncertainty reduction operator indicates that if *u* is close to 0, i.e., *X* has low uncertainty, the weights of original features remain high. If *u* is close to 1, i.e., *X* has high uncertainty, the weights of original features are reduced and should be replaced. Therefore, a novel feature is extracted by a 3 × 3 convolutional layer. The refined feature map $X^{\prime }$ is passed to the next operator.

In this section, a novel fuzzification method is utilized to transform the original convolutional feature maps into the fuzzy domain. Then, uncertainty is computed using fuzzy entropy. New convolutional features and original features are combined to reduce the uncertainties.

### 3.3. Channel-Wise Fuzzy Block

**Figure 4:**
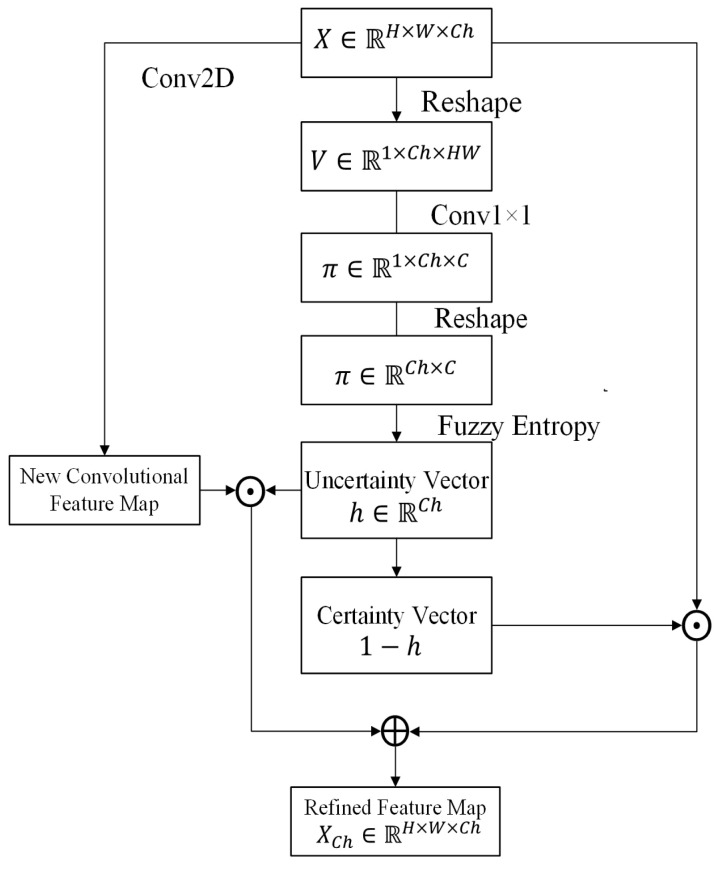
Channel-wise fuzzy block.

After reducing the uncertainty in pixels, the uncertainty in channels is processed by the proposed CFBs. Motivated by the channel-wise attention mechanisms [44,45] and fuzzy logic, the CFB utilizes the fuzzy entropy to measure the uncertainty degree of the channels of feature maps. An uncertain channel is a channel with higher fuzzy entropy (close to 1). There are also three major components in the CFB: fuzzification, uncertainty representation, and uncertainty reduction (Figure 4).

#### 3.3.1. Fuzzification

Let $X\in R^{H\times W\times Ch}$ be the input feature map. *H* and *W* represent the height and width of the feature map, respectively, and Ch is the number of channels. To calculate the uncertainty degree of each channel, it firstly transforms the input feature map into the fuzzy domain in the channel dimension. It reshapes *X* to $V\in R^{HW\times Ch}=\left[v_{1},v_{2},...,v_{Ch}\right]$, where $v_{j}\in R^{HW}$ is the feature vector of channel *j*. For each $v_{j}$, a trainable Sigmoid membership function is utilized to transfer feature vector $v_{j}$ to the fuzzy domain:

(7) $$\pi_{jr}=\frac{1}{1+\exp(\alpha_{jr}v_{j}+\beta_{jr})}$$

where $\pi_{jr}\in R$ represents the membership of category *r* for channel *j*; $\alpha_{jr}\in R^{HW}$ and $\beta_{jr}\in R$ are parameters of channel *j*. The membership is also performed by using two 1×1 convolutional layers with *C* kernels. In order to process *V* using 2D convolutional layer, $V\in R^{HW\times Ch}$ is reshaped to $V\in R^{1\times Ch\times HW}$ before convolutional operators. Then, the convolutional operators are applied:

(8) π=Conv1×1(Conv1×1(V))

where $\pi \in R^{1\times Ch\times C}$ represents the channel membership map for the input feature map *X*; and *C* represents the number of categories. Then, $\pi \in R^{1\times Ch\times C}$ is reshaped to $\pi \in R^{Ch\times C}$ and for each channel, there is a membership vector $\pi_{j}\in \pi =\left[\pi_{j1},\pi_{j2},...,\pi_{jC}\right]$.

#### 3.3.2. Uncertainty Representation

After obtaining the memberships, the fuzzy entropy is computed:

(9) $$h_{j}=-\frac{1}{\log C}\times \sum_{r=1}^{C}\pi_{jr}\log\pi_{jr}$$

where $h_{j}\in R$ represents the fuzzy entropy of channel *j* which measures the uncertainty degree of channel *j*. Finally, the uncertainty degrees $h_{j}$ of all channels in the feature map consist of the uncertainty vector $h\in R^{Ch}=\left[h_{1},h_{2},h_{3},...,h_{j},...,h_{Ch}\right]$.

#### 3.3.3. Uncertainty Reduction

Similar to the SFB, the uncertainty vector *h* is utilized as the weight vector for combining the input feature map and a novel feature map. The novel feature map is generated by a 3×3 convolutional operator. Each element in *h* is the weight value of the corresponding channel:

(10) $$X_{Ch}=\mathrm{Conv}2D\left(X\right)⊙h⊕X⊙\left(1-h\right)$$

where $X_{Ch}\in R^{H\times W\times Ch}$ is the feature map after applying the CFB; ⊙ represents the multiplication between the $j^{\mathrm{th}}$ channel of the feature map and the corresponding scalar $h_{j}$, where j=1,...,Ch. The channel-wise uncertainty reduction operator indicates if *h* is close to 0, the corresponding channels in the input feature map have low uncertainties, and these channels should contain high weights. If *h* is close to 1, i.e., the corresponding channels have high uncertainties. The weights of these channels are reduced, and the input feature should be replaced by a new feature.

### 3.4. Loss Function

The loss function for the proposed SCFURNet can be expressed as the summation of cross entropy and fuzzy entropies from spatial and channel fuzzy blocks:

(11) $$L=L_{c}+L_{s}+L_{Ch}$$

$L_{c}$ is the classic cross-entropy loss function:

(12) $$L_{c}=-\sum_{r}l_{r}\left(x\right)\log\left(p_{r}\left(x\right)\right)$$

where *x* is the input of the network; $l\left(x\right)\in R^{C}=\left[l_{1}\left(x\right),l_{2}\left(x\right),\ldots ,l_{r}\left(x\right),\ldots ,l_{C}\left(x\right)\right]$ is the label of *x* in one-hot encoding. If *x* is in the $r^{\mathrm{th}}$ category, the corresponding $r^{\mathrm{th}}$ element in l(x) is 1 and other elements are 0; $p_{r}\left(x\right)$ represents the proposed network and Soft-max function:

(13) $$p_{r}\left(x\right)=\frac{\exp(a_{r}\left(x\right))}{\sum_{k=1}^{C}\exp\left(a_{k}\left(x\right)\right)}$$

where $a_{r}\left(x\right)$ represents the output of the network; *r* represents the class index, and *C* represents the number of categories.

$L_{s}$ is computed by the fuzzy entropy ($u_{i}$) in the SFBs in Equation (5). Because the SFBs are applied to five convolutional blocks, there are five fuzzy entropy maps from the five convolutional blocks and $L_{s}$ is defined by the summation of fuzzy entropy maps.

(14) $$L_{s}=\sum_{l}\sum_{i}u_{i}^{l}$$

where *i* represents the pixel index and *l* represents the index of convolutional blocks.

$L_{Ch}$ is computed by the fuzzy entropy ($h_{j}$ in Equation (9)) in CFBs.

(15) $$L_{Ch}=\sum_{l}\sum_{j}h_{j}^{l}$$

where *j* represents the channel index.

The loss terms $L_{Ch}$ and $L_{s}$ are the uncertainty degrees in five spatial and channel-wise fuzzy blocks. Adding these two loss terms can minimize the classification loss and uncertainty in pixel and channel dimensions simultaneously to obtain less uncertainty feature maps. The error propagates using a standard back-propagation algorithm [55].

## 4. Experimental Results

### 4.1. Datasets

To show the effectiveness of the proposed network in BUS image semantic segmentation, two kinds of experiments are designed: (1) multi-object (multi-layer) semantic segmentation, and (2) binary semantic segmentation (tumor and background). The multi-object semantic segmentation is performed on a dataset having 325 BUS images. The dataset is collected by the Second Affiliated Hospital of Harbin Medical University and the First Affiliated Hospital of Harbin Medical University. An experienced radiologist from the First Affiliated Hospital of Harbin Medical University delineate the boundaries of the four breast layers and tumors. The privacy of the patient is well protected. The pixel-wise ground truths for five categories: fat layer, mammary layer, muscle layer, tumor, and background are generated according to the manually delineated boundaries. In multi-object semantic segmentation task, the proposed method is compared with state-of-the-art deep learning segmentation methods such as U-Net with VGG-16 [46], U-Net with ResNet-50/ResNet-101 [47], Deeplab [56], FCN-8s [36], PSPNet [57], and U-Net with information extension [16]. We also compare the proposed methods with some spatial and channel-wise attention modules such as attention U-Net [43], SE-Net [44], and self-attention mechanism [58].

The binary semantic segmentation is performed on three public BUS image datasets [35,59,60]. Dataset [48] contains 163 BUS images including 109 benign samples and 54 malignant samples. Dataset [60] contains 780 BUS images including 437 benign, 210 malignant, and 133 no tumor images. Ref. [59] is a BUS image benchmark with 562 images and lists five non-deep learning methods [10,61,62,63,64] for BUS image segmentation. In this task, state-of-the-art semantic segmentation network structures are also applied for comparison. Also, five traditional tumor segmentation methods [10,61,62,63,64] are utilized for comparison. The summary of the four datasets used in experiments is listed in Table 1.

### 4.2. Experiment Details

#### 4.2.1. Preprocessing and Augmentation

Because of the number limitation of samples, the training samples are augmented by horizontal flip, horizontal shift, vertical shift, rotation, zooming, and shear mapping. The input images are all gray-level images and intensities are mapped into [−1, 1] by (x/127.5−1) [65], where *x* represents the original intensity. No other augmentation methods are used except U-Net with information extension [16]. In [16], the input images are firstly preprocessed by histogram equalization. Then, images are transformed into the wavelet domain. New three-channel images with grey-level intensity in the first channel, wavelet approximation coefficients in the second channel, and wavelet detail coefficients in the third channel are utilized for training the original U-Net with ResNet-101 network.

#### 4.2.2. Experiment Environment

All the networks in this section are not pre-trained using other datasets. The network weights are initialized randomly. The input image is resized to 128×128. The batch size is 12. The optimizing method is the stochastic gradient descent (SGD) method, with a learning rate of 0.001 and momentum of 0.99. The training epoch number is 80. All the comparison networks and the proposed method are trained using a computer with Ubuntu 18.04 system, Intel (R) Xeon (R) CPU E5-2620 2.10GHz and 8 NVIDIA GeForce 1080 graphics cards, and each one has 8 Gigabyte memory. The implementation uses PyTorch 1.6.0.

### 4.3. Metrics

In binary semantic segmentation task, it utilizes metrics in [59] to evaluate the performance. There are five area metrics: true positive ratio (TPR), false positive ratio (FPR), Jaccard index (JI), Dice’s coefficient (DSC), and area error ratio (AER). The area metrics are defined in the following equation:

(16) $$\begin{array}{cc} \mathrm{TPR}= & |A_{r}\cap A_{m}\left|/\right|A_{m}|\\ \mathrm{FPR}= & |A_{r}\cup A_{m}-A_{m}\left|/\right|A_{m}|\\ \mathrm{JI}= & |A_{r}\cap A_{m}\left|/\right|A_{r}\cup A_{m}|\\ \mathrm{DSC}= & 2|A_{r}\cap A_{m}\left|/\right|A_{r}\left|+\right|A_{m}|\\ \mathrm{AER}= & \left(\right|A_{r}\cup A_{m}\left|-\right|A_{r}\cap A_{m}\left|)/\right|A_{m}| \end{array}$$

where $A_{r}$ is the set of pixels generated by the proposed method or existing methods, and $A_{m}$ is the set of pixels in the ground truths.

In the multi-object semantic segmentation task, intersection over union (IoU, also known as the Jaccard index in the binary task) is a typical metric in semantic segmentation and is chosen as the metric here. It is computed by:

(17) $$\mathrm{IoU}=|A_{r}\cap A_{m}\left|/\right|A_{r}\cup A_{m}|$$

where $A_{r}$ and $A_{m}$ are the sets of pixels generated by the algorithms and ground truths, respectively. Mean IoU (mIoU=∑IoU/C, and *C* represents the number of categories) over five categories to evaluate the overall performance.

### 4.4. Multi-Object Semantic Segmentation of BUS Images

**Figure 5:**
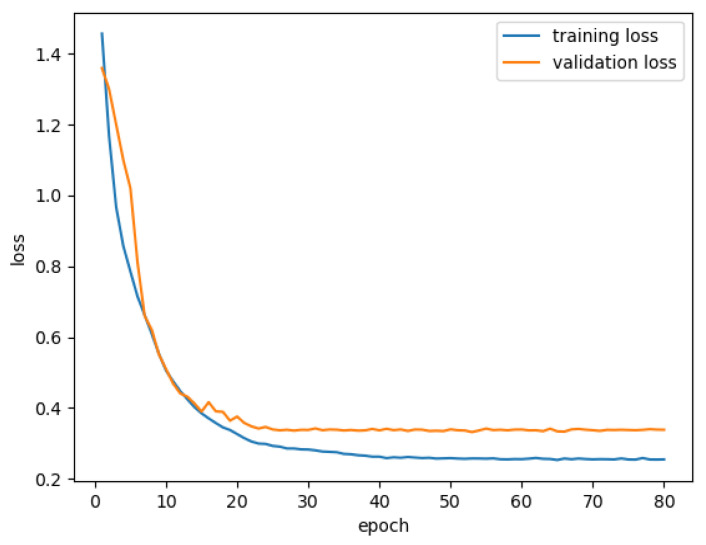
Training and validation loss curves for m1ulti-object semantic segmentation of BUS images.

In this section, we discuss the performance of SCFURNet on the multi-layer dataset. We first present segmentation results of SCFURNet with different numbers of fuzzy blocks (SFBs and CFBs); next explain ablation study for the proposed fuzzy blocks; then visualize uncertainty maps obtained by fuzzy blocks; finally discuss the quantitative semantic segmentation results of SCFURNet and all compared methods. The dataset with 325 BUS images is utilized, and each of them contains pixel-wise ground truths of five categories. 10-fold validation is also utilized. The proposed SFBs and CFBs are applied to U-Net with VGG-16/ResNet-101 as the encoder. The training and validation loss curve is shown in Figure 5. The loss is calculated based the average of 10-fold validation.

#### 4.4.1. Segmentation Performance and the Number of Fuzzy Blocks

In this subsection, we discuss the relation between the number of fuzzy blocks used in the network and the performance of the segmentation. The U-Net with ResNet-101 is utilized in this research. The proposed SFB and the CFB are applied to the encoder of the U-Net with ResNet-101. The ResNet-101 contains 5 convolutional blocks; therefore, we use 5 fuzzy blocks as the maximum number to conduct experiments for comparison. In the first experiment, there is no fuzzy block applied to the U-Net with ResNet-101. In the second experiment, the proposed spatial and channel-wise fuzzy blocks are applied to the first convolutional block. We continue adding the spatial and channel-wise fuzzy blocks to the second, third, fourth, and fifth convolutional blocks and keeping the fuzzy blocks in the previous convolutional blocks.

**Figure 6:**
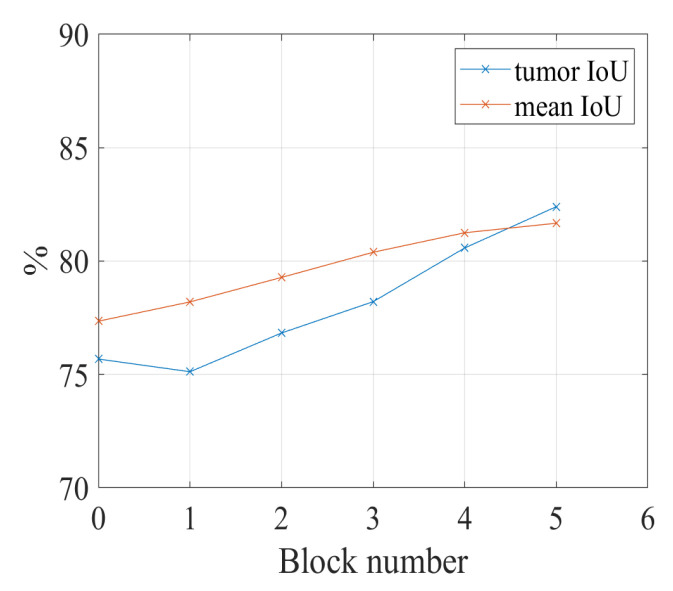
The relation between the number of fuzzy blocks and the segmentation performance. Block number = 1: the fuzzy blocks are applied to the first convolutional block; block number = 2: the fuzzy blocks are applied to the first and second convolutional blocks together; block number = 3: the fuzzy blocks are applied to the convolutional blocks 1, 2, and 3; block number = 4: the fuzzy blocks are applied to the convolutional blocks 1, 2, 3, and 4; block number = 5: the fuzzy blocks are applied to the convolutional blocks 1, 2, 3, 4, and 5. The reason for the maximum number of blocks is 5 will be given in Section 4.4.1.

Figure 6 shows IoU results vs. the number of convolutional blocks. When we apply the spatial and channel-wise fuzzy blocks to all five convolutional blocks, the proposed network achieves the best performance on both tumor category and the overall performance.

**Figure 7:**
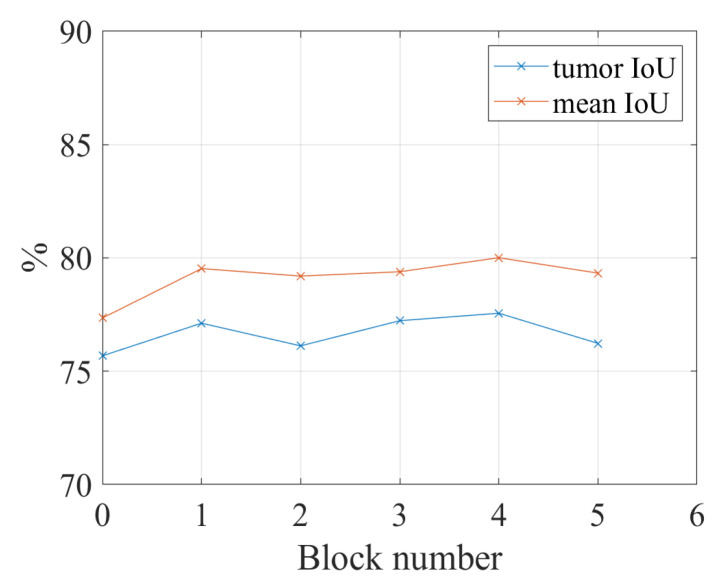
The relation between the number of fuzzy blocks and the segmentation performance. The fuzzy blocks are applied to the convolutional blocks individually. Block number = 1: the fuzzy blocks are applied to the first convolutional block; block number = 2: the fuzzy blocks are applied to the second convolutional block; block number = 3: the fuzzy blocks are applied to the third convolutional block; block number = 4: the fuzzy blocks are applied to the fourth convolutional block; block number = 5: the fuzzy blocks are applied to the fifth convolutional block.

In order to show the increasing performance in Figure 6 is caused by the fuzzy blocks in deeper convolutional blocks or the combination of the former fuzzy blocks and the newly added fuzzy blocks, another experiment is conducted. In this experiment, the fuzzy blocks are added to the five convolutional blocks of ResNet-101 individually. For example, the fuzzy blocks are added to the second convolutional block of ResNet-101; there is no fuzzy block in convolutional blocks 1, 3, 4, and 5. The experiment results in Figure 7 show that there is a slight increase in performance when applying fuzzy blocks to convolutional blocks 1 to 5; however, the performance cannot outperform the performance of using fuzzy blocks in five convolutional blocks together. When we only add a fuzzy block to the fourth convolutional block, the IoU for the tumor is the highest, which is 77.56%; however, when we add fuzzy blocks to all five convolutional blocks, the IoU for the tumor is 82.40%. Therefore, the spatial and channel-wise fuzzy blocks are applied to five convolutional blocks in the following experiments.

#### 4.4.2. Ablation Study for Fuzzy Blocks

We employed the SFB and the CFB in five convolutional blocks to reduce the uncertainty in the feature maps. To verify the performance of each fuzzy block, we conduct experiments with different settings in Table 2.

As shown in Table 2, it compares two convolutional structures: VGG-16 and ResNet-101. Meanwhile, it adopts the SFB and the CFB individually in each network. Compared with the U-Net with VGG-16, employing the SFB brings a 1.94% increase in tumor IoU and 2.41% in mean IoU. Meanwhile, employing the CFB in U-Net with VGG-16 outperforms the baseline by 0.97% in tumor IoU and 3.68% in mean IoU. When the two fuzzy blocks are used together to the U-Net with VGG-16, the performance further improved to 78.34% in tumor IoU and 79.36% in mean IoU. When changing the convolutional structure to ResNet-101, the performance of using two fuzzy blocks together becomes 82.40% in tumor IoU and 81.67% in mean IoU. Here we choose to show the tumor segmentation results and overall performance because tumors are the most important object in BUS image segmentation. The experiment results show that each fuzzy block can reduce uncertainty in the feature maps and increase the performance of tumor segmentation.

**Figure 8:**
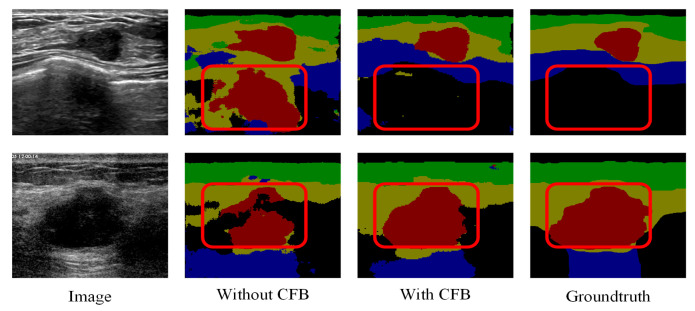
Segmentation results of U-Net with ResNet-101 and CFB on multi-object dataset. Green: fat layer, yellow: mammary layer, blue: muscle layer, red: tumor, and black: background. The red rectangles represent the mis-segmented regions by the baseline module.

**Figure 9:**
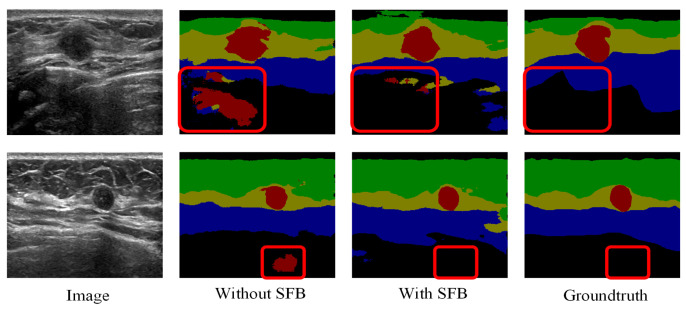
Segmentation results of U-Net with ResNet-101 and SFB on multi-object dataset. Green: fat layer, yellow: mammary layer, blue: muscle layer, red: tumor, and black: background. The red rectangles represent the mis-segmented regions by the baseline module.

The effectiveness of the proposed channel and spatial-wise fuzzy blocks can be shown in Figure 8 and Figure 9, respectively. The most common misclassification is the tumor area and the background area because both areas contain low intensities. The misclassification patches are marked by red rectangles in Figure 8 and Figure 9. They are correctly classified when applied the SFB or CFB individually.

#### 4.4.3. Visualization of Fuzzy Blocks

In this part, the uncertainty maps obtained by the SFB and selected channels in the processed feature maps are visualized for a better understanding of the proposed SFB and the CFB.

**Figure 10:**
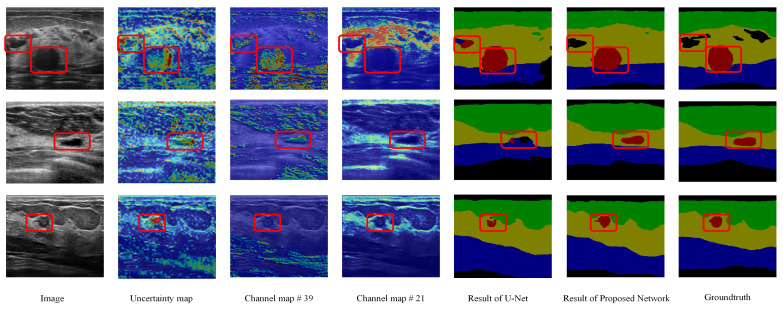
Visualization results of fuzzy blocks on the multi-object dataset. For each row, we show an input image, an uncertainty map from the SFB; red represents a high value and blue represents a low value in the heatmap. We also provide two channel maps from the outputs of the CFB, the results of the original U-Net and the proposed method, and the groundtruths. Green: fat layer, yellow: mammary layer, blue: muscle layer, red: tumor, and black: background. The red rectangles represent the mis-segmented regions by the baseline module or tumor regions.

The SFB is utilized to measure the uncertainty degree of pixels in the input feature map and reduce the effect of the uncertain pixels. Therefore, the uncertainty map generated in the SFB can show the uncertain pixels and corresponding uncertainty degrees (refer to Figure 10). For example, the areas marked by red rectangles are background and tumor areas in the first row. They have similar intensities. In the uncertainty map, these areas are high uncertainty areas. The original U-Net misclassifies the background area; however, the proposed method can correct it (shown in columns 5 and 6). In the second row and third row, the tumor areas are also marked as the uncertain areas, i.e., the original U-Net cannot handle these areas. The heatmaps indicate that the proposed SFB can find the uncertain areas of the input feature maps, and it can also measure the uncertainty degree of the pixels.

For CFB, it is hard to give a comprehensible visualization about the uncertainty map directly because each channel of the input feature map only contains an uncertainty value. Instead, we show some processed channels to see whether they highlight clear semantic areas. In Figure 10, we display the 39th and 21st channels of each feature map after employing a CFB. We can see that in the 21st channel of the feature map, the highlighted areas are in the mammary layers. The 39th channel of the feature map highlights the area of the tumor. However, some areas in other categories contain high response in the 39th channel of the feature maps as well (such as the muscle layer in the first and third rows and the fat layer in the second row). These results indicate that the proposed fuzzy blocks can help generate feature maps with clear semantic information.

#### 4.4.4. Semantic Segmentation Results

**Figure 11:**
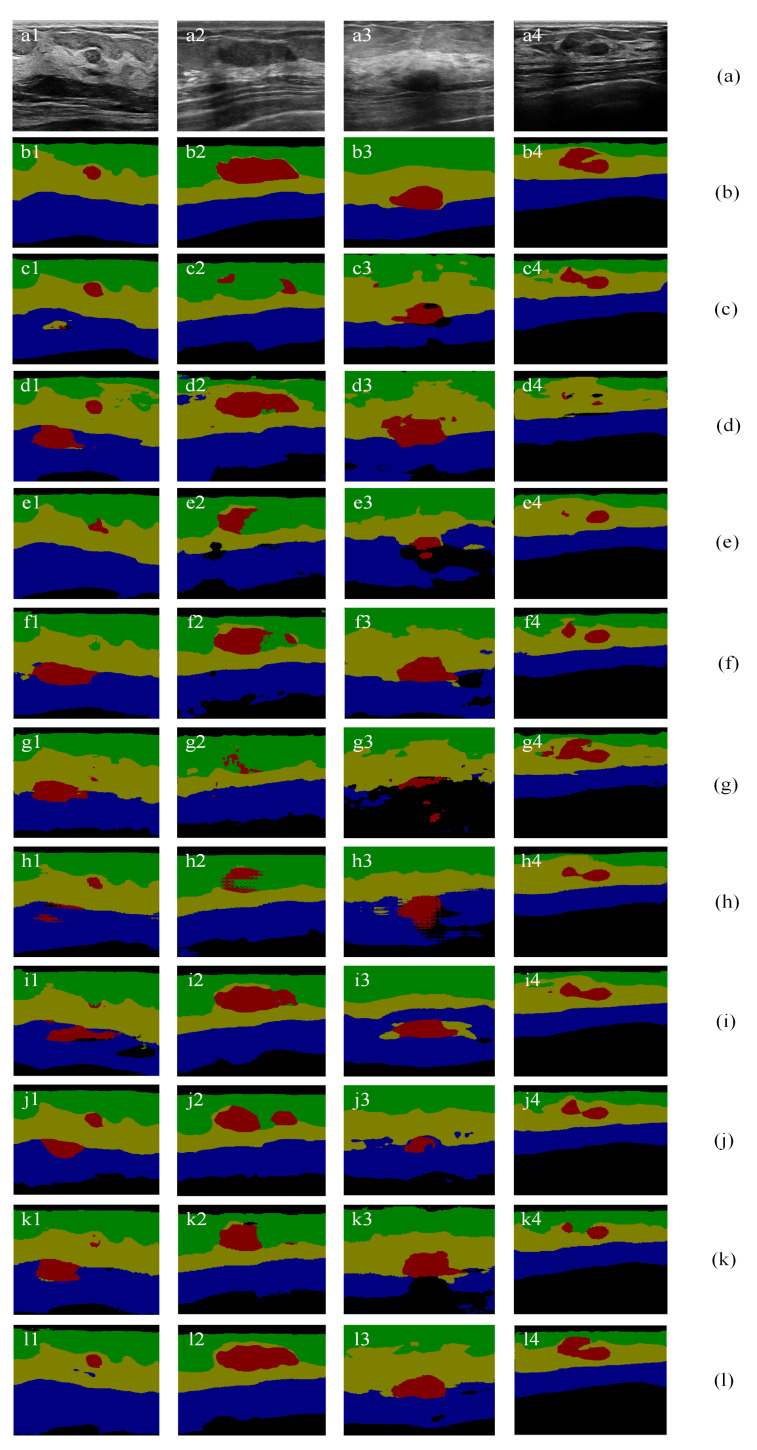
Multi-object semantic segmentation of BUS images: (**a**) original images; (**b**) ground truths; (**c**) results of ResNet-101 + self-attention mechanism; (**d**) results of attention U-Net; (**e**) results of ResNet-50; (**f**) results of ResNet-101; (**g**) results of Deeplab; (**h**) results of PSPNet; (**i**) results of U-Net with wavelet transform; and (**j**) results of FCN-8s; (**k**) results of SE-Net (ResNet-101); (**l**) results of the proposed method.

Figure 11 illustrates segmentation results of SCFURNet and nine compared methods for four representative BUS images in the multi-object datasets. Figure 11b shows the pixel-wise ground truths: the green areas are fat layers; the yellow areas are mammary layers; the blue areas are muscle layers; the red areas are tumors, and the black areas are background areas.

The results in Figure 11i are obtained when the input images are the three-channel images with gray-level intensity in the first channel, wavelet approximation coefficients in the second channel, and wavelet detail coefficients in the third channel and the network structure is the U-shape network with ResNet-101. The results in Figure 11f are obtained when the images are the original gray-level images, and the network structure is the same as the network used in Figure 11i. Comparing Figure 11i and Figure 11f, the tumor segmentation results in Figure 11i2,i4 are better than that in Figure 11f2,f4. However, the results in Figure 11i1,i3 are not improved. The experiment results of using wavelet feature in the input layer prove that involving wavelet feature cannot handle some misclassification such as the background area and tumor area because they contain similar feature values in both wavelet domain and space domain.

The proposed method generates new convolutional features. New convolutional feature maps and original convolutional feature maps are combined using uncertainty degree as the weights in pixels and channels. It reduces the effect of uncertain pixels and uncertain channels. This mechanism overcomes the drawback in Figure 11i. For example, in Figure 11f3, the original U-Net with ResNet-101 can segment the tumor. In Figure 11i3, when adding wavelet features, the segmentation results of tumors and the mammary layer become worse. Other network structures also do not handle these images well. The quantitative results of Figure 11 show that the proposed method improves the second-best method significantly by 2.45%, 3.38%, and 14.36% for the mIoU for Figure 11a1–a3. The overall mIoU only improves 0.53% for Figure 11a4; however, the proposed method improves the second-best method by 11.71% for tumor IoU. The performances are shown in Table 3. Bold numbers represent the corresponding best results. The IoU increases 6.72% in tumor segmentation compared with that of the original U-Net with ResNet-101. It achieves a 7.52% improvement in IoU in tumor segmentation compared with that of the U-Net with ResNet-101 and wavelet transform. The proposed method achieves 4.32% and 4.05% improvements in overall mIoU compared with that of the U-Net with gray-level intensity and wavelet transform, respectively. The proposed method achieves the best performance in tumor segmentation and the best overall performance among all methods. The overall performance indicates that the proposed method can handle misclassification caused by similar feature values of different layers because the proposed method can reduce the weights of the similar features of different layers and add novel features.

### 4.5. Semantic Segmentation on Three Public Two-Category Datasets

We also conduct experiments on three two-category public datasets to evaluate the performance of SCFURNet on the binary segmentation (tumor and background) task.

#### 4.5.1. Overall Performance on Three Public Datasets

The proposed SFB and CFB are applied to a U-Net with ResNet-101 network because it achieves better results compared with U-Net with VGG-16 in Section 4.4.2. All other compared deep networks such as ResNet-50, ResNet-101, and FCN-8s are trained to segment tumors in these three datasets. Because of the limited number of samples (the total number of samples for 3 datasets is only 1505), 10-fold validation is utilized: (1) each of the three datasets is divided into 10 groups randomly; (2) pick 9 groups of each dataset as the training set and the rest 1 group as the testing set; and (3) the final evaluation metrics are calculated by the average of 10 experiments.

**Figure 12:**
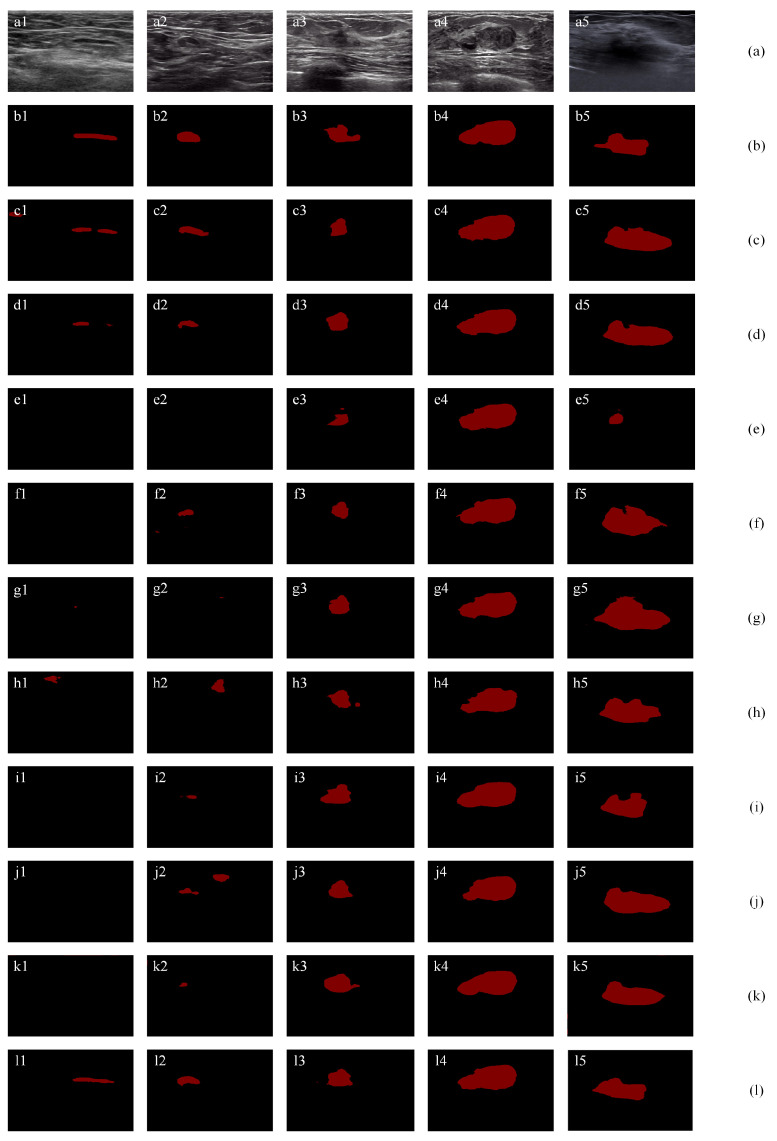
Segmentation results using public dataset: (**a**) original images; (**b**) ground truths; (**c**) results of ResNet-101 with self-attention mechanism; (**d**) results of a SE-Net (ResNet-101); (**e**) results of attention U-Net; (**f**) results of ResNet-50; (**g**) results of ResNet-101; (**h**) results of Deeplab; (**i**) results of PSPNet; (**j**) results of U-Net with wavelet transform; and (**k**) results of FCN-8s; (**l**) results of proposed method.

Figure 12 shows the segmentation results using the three two-category datasets [35,59,60]. Figure 12 (a) shows the original images and (b) shows the ground truths. For Figure 12a1 containing a narrow and long tumor, most methods (e1, f1, g1, i1, and j1) fail to segment the tumor; h1 mistakenly segments a wrong tumor region; c1 and d1 segment the tumor region with small JI values of 41.65% and 17.61%, respectively; the proposed method l1 achieves the highest JI value of 55.19%. For Figure 12a2, most methods (e2, f2, g2, h2, i2, j2, and k2) fail to segment the tumor or mistakenly segment a wrong tumor region since their JI values are less than 26%. For three methods (c2, d2, and l2) correctly segment the tumor region, the proposed method (l1) achieves the highest JI value of 88.38%, which significantly outperforms c2 (44.28%) and d2 (35.01%). For Figure 12a3 containing an irregular tumor, the proposed method (l3) achieves the highest TPR, JI, DSC, and the lowest AER values. Specifically, it outperforms the second-best method by 2.78%, 15.41%, 8.55%, and 35.02% for TPR, JI, DSC, and AER, respectively. For Figure 12a4 containing a big tumor, all methods achieve good segmentation results. The proposed method (l3) achieves the highest TPR, JI, and DSC values of 97.57%, 93.17%, and 96.47%, and the lowest FPR and AER value of 4.72% and 7.15%. For Figure 12a5 containing an irregular tumor with unclear contour, the proposed method (l3) achieves the best segmentation results with the highest JI of 84.86%, the highest DSC of 91.81% and the lowest AER of 17.30%.

Table 4 summarizes segmentation results of SCFURNet, 9 deep learning methods, and 5 classic machine learning methods in terms of five measures on the dataset [59]. Five non-deep learning methods [10,61,62,63,64] are also involved in the comparison using this dataset. Results in Table 4 show: (1) deep learning methods obtain improvements compared with traditional BUS image segmentation methods listed in [59]; (2) some famous deep learning architectures such as Deeplab, PSPNet, do not obtain improvements for dataset [59] and the possible reason is the limited number of the samples; and (3) the proposed method achieves the best results since it can solve the small target problems and uncertainties in the boundary areas.

Table 5 summarizes segmentation results of SCFURNet and 9 peer deep learning methods on Dataset [35] and Dataset [60]. The proposed method achieves the best results among all evaluation metrics compared with state-of-the-art deep learning methods on three public datasets except the FPR and AER on dataset [35]. The self-attention mechanism in ResNet-101 obtains lower FPR and AER on dataset [35]. Lower FPR and AER indicate that non-local context information provided by the self-attention mechanism can help to reduce errors in segmentation. However, the proposed method achieves the best overall performance by reducing uncertainty in pixels and channels. The proposed method achieves 2.03%, 1.84%, and 2.88% in the Jaccard index on three public BUS datasets compared with that of the original U-shape network with ResNet-101, respectively.

#### 4.5.2. Small Tumor Segmentation

**Figure 13:**
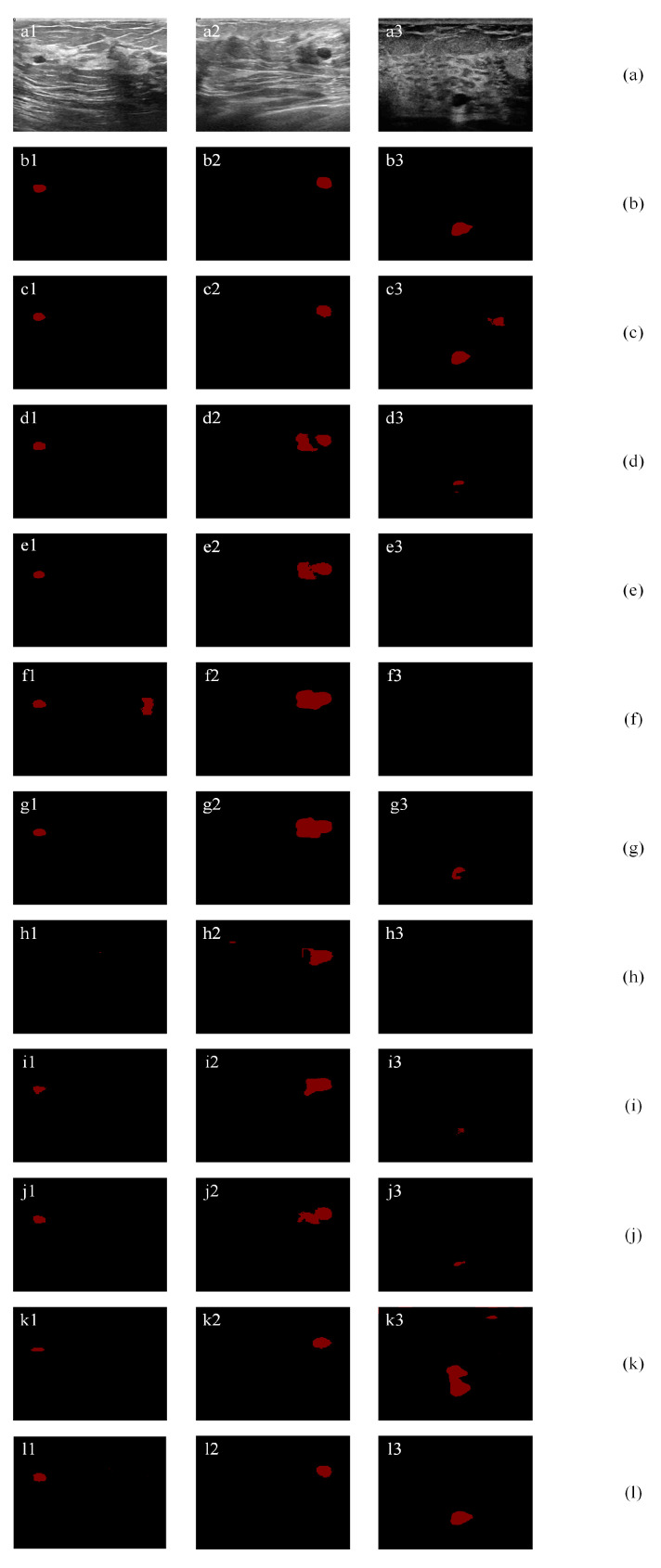
Small tumor segmentation: (**a**) original images; (**b**) ground truths; (**c**) results of ResNet-101 with self-attention mechanism; (**d**) results of a SE-Net (ResNet-101); (**e**) results of attention U-Net; (**f**) results of ResNet-50; (**g**) results of ResNet-101; (**h**) results of Deeplab; (**i**) results of PSPNet; (**j**) results of U-Net with wavelet transform; and (**k**) results of FCN-8s; (**l**) results of proposed method.

In this section, we show the effectiveness of the proposed method on small tumor segmentation. Small tumors are hard to segment due to their small size, low intensity, and tumor-like regions in BUS images. Figure 13a1 contains a very small tumor. The proposed method (l1) achieves the highest TPR, JI, and DSC values of 92.78%, 81.82%, and 90.00%, and the lowest AER value of 20.62%. It improves the second-best method by 1.12%, 1.78%, 0.98%, and 0.54% in terms of TP, JI, DSC, and AER, respectively. Figure 13a2 contains a small tumor close to a tumor-like region. Most existing methods mistakenly classify the tumor-like region. The proposed method (l1) achieves the highest JI and DSC values of 85.11% and 91.95%, and the lowest AER value of 16.91%. It improves the second-best method by 5.16%, 2.78%, and 14.64% in terms of JI, DSC, and AER, respectively. Figure 13a3 contains a small tumor that is located in a region in low intensity, which makes it hard to be distinguished from the background. The proposed method (l1) achieves the highest JI and DSC values of 70.63% and 82.79%, and the lowest AER values of 39.50%. It improves the second-best method by 9.75%, 5.71%, and 17.16% in terms of JI, DSC, and AER, respectively. It is due to the fact that the small tumor contains similar feature values with noise patches or background patches. However, the proposed method achieves the best results in small tumor images; therefore, it can achieve the best overall performance on all datasets.

## 5. Discussion

### 5.1. Comparison with Previous Studies and Potential Usefulness

We propose a novel SCFURNet with SFBs and CFBs to reduce the uncertainty in convolutional feature maps. The proposed method can perform a anatomy segmentation on BUS images. In general, four significant advantages of the proposed network surpasses previous BUS image segmentation methods.

First, the proposed SFBs and CFBs are individual blocks that do not depend on network structures. They can be easily integrated into different network structures, such as VGG-16 and ResNet. Most other attention mechanisms are either designed with new network structures or have limitations when applied to other networks. Second, as shown in Table 2, the proposed SFBs and CFBs can be used with different networks, and removing either block will lead to worse performance on BUS image segmentation. That is because the SFBs and CFBs can both find the fuzzy regions and channels in the feature maps and reduce their weights, refers to Figure 10. Third, our SFBs and CFBs can also be used in the semantic segmentation of other datasets besides BUS images. Fourth, in Section 4.5.2, we prove that the proposed SFBs and CFBs can help detect small tumor regions. Small tumors and low-intensity background regions have high uncertainty degrees (Figure 10). We focus on those uncertain regions and refine their feature maps to get better segmentation results.

Potential usefulness: the proposed SCFURNet can be applied to build trustworthy ultrasound image CAD systems from a clinical perspective. The propsed method can be used in splitting the BUS images into breast layer structures. It is helpful to diagnosis benign and malignant tumors in BUS images in clinical applications. We also explain the attention mechanism based on fuzzy logic and uncertainty, while existing attention methods are based on statistics and probability.

### 5.2. Limitations

While the proposed SCFURNet can measure the uncertainty and refine convolutional feature maps to get better segmentation results, there are some limitations. First, the proposed method is based on other supervised deep learning networks, which means we still need to use pixel-wise ground truths to train the SCFURNet to classify five classes on BUS images. The labor cost of generating pixel-wise ground truths is high. Second, the evaluation of the proposed method is limited. Due to the fact that we only have a limited number of samples for training and validation, we use the 10-fold cross-validation method in the experiment section. There is no independent test set, which means our experiment results might overfit to specific datasets and the generalizability of the proposed method is untested.

## 6. Conclusions

In this paper, we design a trustworthy SCFURNet for BUS image semantic segmentation. SCFURNet consists of two kinds of fuzzy blocks: spatial-wise fuzzy blocks (SFBs) and channel-wise fuzzy blocks. The proposed method can segment five breast layer structures of BUS images. The proposed SCFURNet achieves 2.03%, 1.84%, and 2.88% improvements in the Jaccard index using three public BUS datasets compared with that of the original U-shape network with ResNet-101. SCFURNet also improves the original U-shape network with ResNet-101 by 6.72% for tumor IoU and by 4.32% for mean IoU in the five-category BUS dataset.

SCFURNet achieves the best results due to the following reasons: (1) The proposed spatial and channel-wise fuzzy blocks can locate uncertain pixels and uncertain channels in feature maps and can reduce the influence of uncertain pixels and channels; (2) By reducing the uncertainty in feature maps, some patches having similar features with that of tumor areas can be classified correctly, especially for small tumors; (3) The fuzzy entropy of memberships can measure the uncertainty degree of pixels and channels accurately. The experimental results validate the following claims: (1) there are uncertainty and noise in BUS images, especially for small tumors and background areas; (2) the proposed method can reflect the uncertain pixels and uncertain channels and generate better feature maps; and (3) the proposed method can solve small target problem.

In the future, we plan to explore novel methods to extract more certain features which directly have low fuzzy entropy compared with convolutional operators. We also plan to develop different uncertainty representation methods and compare them with fuzzy entropy. Another research direction is designing weakly supervised method to reduce the labor cost in ground truth generation. Finally, we will try to extend the proposed network to other image segmentation dataset with more training samples, such as the nuclei image classification and segmentation dataset, PanNuke [66].

## Figures and Tables

**Table 1:** Dataset Properties.

	Image Number	Ground Truths
Dataset 1 [35]	163	Tumor/Background
Dataset 2 [60]	780	Tumor/Background
Dataset 3 [59]	562	Tumor/Background
Multi-layer Dataset	325	Fat/Mammary/Muscle/Tumor/Background

**Table 2:** Ablation Study on Multi-object Dataset. SFB: Spatial-wise Fuzzy Block, CFB: Channel-wise Fuzzy Block.

Encoder	SFB	CFB	Tumor IoU	Mean IoU
VGG-16			74.66%	75.13%
VGG-16	✓		76.60%	77.54%
VGG-16		✓	75.63%	78.81%
VGG-16	✓	✓	78.34%	79.36%
ResNet-101			75.68%	77.35%
ResNet-101	✓		79.12%	78.67%
ResNet-101		✓	80.43%	80.12%
ResNet-101	✓	✓	**82.40%**	**81.67%**

Bold numbers are the corresponding best results. Checkmarks denote the SFB or CFB added to the baselines.

**Table 3:** Results of Multi-object Semantic Segmentation. Evaluation Metric is IoU (%).

	Fat	Mammary	Muscle	Background	Tumor	Mean
ResNet-50	82.58	73.98	73.08	77.23	76.34	76.64
ResNet-101	82.50	74.41	75.69	77.47	75.68	77.35
FCN-8s	82.57	75.47	75.53	78.59	74.42	77.32
PSPNet	82.07	74.40	74.49	77.36	74.75	76.61
Deeplab	78.91	68.71	67.33	73.94	69.04	71.58
Attention U-Net	83.99	77.61	75.69	77.99	76.26	78.31
SE-Net	80.91	75.21	71.23	76.57	75.90	75.96
Self-attention	82.53	76.23	75.91	80.29	78.81	78.75
[16]	84.05	75.92	74.89	78.35	74.88	77.62
Proposed	**84.72**	**79.84**	**77.39**	**83.98**	**82.40**	**81.67**

Bold numbers are the best results.

**Table 4:** Results of Two-class Semantic Segmentation on Dataset [59].

	TPR	FPR	JI	DSC	AER
	Semi-Automatic Methods				
[10]	0.82	0.13	0.73	0.84	0.31
[64]	0.84	0.07	0.79	0.88	0.23
	Fully-Automatic Methods				
[61]	0.81	0.16	0.72	0.83	0.36
[62]	0.81	1.06	0.60	0.70	1.25
[63]	0.67	0.18	0.61	0.71	0.51
Deeplab	0.89	0.11	0.82	0.89	0.22
ResNet50	0.92	0.08	0.86	0.92	0.16
ResNet101	0.92	0.10	0.85	0.91	0.18
FCN8s	**0.94**	0.10	0.86	0.92	0.16
PSPNet	0.93	0.09	0.86	0.92	0.16
Attention U-Net	0.92	0.09	0.85	0.91	0.17
SE-Net	0.92	0.10	0.85	0.91	0.18
Self-attention	0.91	0.07	0.86	0.92	0.15
[16]	0.92	0.09	0.86	0.92	0.16
Proposed	**0.94**	**0.06**	**0.88**	**0.93**	**0.14**

Bold numbers are the best results.

**Table 5:** Results of Two-class Semantic Segmentation on Dataset [35] and Dataset [60].

		TPR	FPR	JI	DSC	AER
Dataset [35]	Deeplab	63.68%	36.06%	52.93%	61.91%	72.38%
	ResNet50	81.29%	36.58%	68.70%	76.94%	55.29%
	ResNet101	83.58%	34.40%	71.43%	79.45%	50.82%
	FCN8s	82.72%	41.14%	67.50%	76.87%	58.42%
	PSPNet	81.08%	40.42%	69.77%	78.24%	59.34%
	Attention-UNet	83.58%	34.40%	71.43%	79.45%	50.82%
	Self-attention	82.58%	**26.39%**	73.83%	81.37%	**33.81%**
	SE-Net	79.23%	36.75%	70.90%	79.10%	35.12%
	[16]	81.19%	31.63%	71.48%	80.21%	48.44%
	Proposed	**84.70%**	44.69%	**73.27%**	**81.08%**	59.99%
Dataset [60]	Deeplab	59.88%	39.39%	49.65%	59.39%	79.52%
	ResNet50	78.45%	49.39%	67.09%	76.36%	68.94%
	ResNet101	79.40%	46.02%	69.26%	77.90%	66.62%
	FCN8s	74.23%	46.69%	63.16%	73.03%	72.63%
	PSPNet	77.11%	46.65%	65.21%	74.75%	69.54%
	Attention-UNet	77.52%	38.67%	67.81%	76.77%	60.92%
	Self-attention	79.02%	29.30%	71.49%	78.46%	55.50%
	SE-Net	78.40%	38.95%	68.30%	77.24%	60.55%
	[16]	78.07%	42.37%	68.43%	76.96%	64.30%
	Proposed	**79.86%**	**22.01%**	**72.14%**	**80.51%**	**42.15%**

Bold numbers are the best results.

## Data Availability

Publicly available datasets were analyzed in this study. Datasets can be found here: Dataset 1 [35], link: http://www2.docm.mmu.ac.uk/STAFF/m.yap/dataset.php, Dataset 2 [60], link: https://scholar.cu.edu.eg/?q=afahmy/pages/dataset, Dataset 3 [59], link: http://cvprip.cs.usu.edu/busbench/. Data sharing is not applicable for Multi-layer Dataset.
